# Performance status (PS) as a predictor of poor response to immune checkpoint inhibitors (ICI) in recurrent/metastatic head and neck cancer (RMHNSCC) patients

**DOI:** 10.1002/cam4.4722

**Published:** 2022-03-29

**Authors:** Cameron Chalker, Jenna M. Voutsinas, Qian Vicky Wu, Rafael Santana‐Davila, Victoria Hwang, Christina S. Baik, Sylvia Lee, Brittany Barber, Neal D. Futran, Jeffrey J. Houlton, George E. Laramore, Jay Justin Liao, Upendra Parvathaneni, Renato G. Martins, Keith D. Eaton, Cristina P. Rodriguez

**Affiliations:** ^1^ Department of Medicine University of Washington Seattle Washington USA; ^2^ Clinical Research Division Fred Hutchinson Cancer Research Center Seattle Washington USA; ^3^ Division of Medical Oncology, Department of Medicine University of Washington Seattle Washington USA; ^4^ Department of Obstetrics and Gynecology John Peter Smith Hospital Fort Worth Texas USA; ^5^ Department of Otolaryngology Head and Neck Surgery University of Washington Seattle Washington USA; ^6^ Department of Radiation Oncology University of Washington Seattle Washington USA

**Keywords:** checkpoint control, clinical cancer research, clinical management, head and neck cancer, immunology, prognostic factor

## Abstract

**Background:**

Anti‐PD1 checkpoint inhibitors (ICI) represent an established standard‐of‐care for patients with recurrent/metastatic head and neck squamous cell carcinoma (RMHNSCC). Landmark studies excluded patients with ECOG performance status (PS) ≥2; the benefit of ICI in this population is therefore unknown.

**Methods:**

We retrospectively reviewed RMHNSCC patients who received 1+ dose of ICI at our institution between 2013 and 2019. Demographic and clinical data were obtained; the latter included objective response (ORR), toxicity, and any unplanned hospitalization (UH). Associations were explored using uni‐ and multivariate analysis. Overall survival (OS) was estimated using a Cox proportional hazards model; ORR, toxicity, and UH were evaluated with logistic regression.

**Results:**

Of the 152 patients, 29 (19%) had an ECOG PS ≥2. Sixty‐six (44%) experienced toxicity; 54 (36%) had a UH. A multivariate model for OS containing PS, smoking status, and HPV status demonstrated a strong association between ECOG ≥2 and shorter OS (*p* < 0.001; HR = 3.30, CI = 2.01–5.41). An association between OS and former (vs. never) smoking was also seen (*p* < 0.001; HR = 2.17, CI = 1.41–3.35); current smoking did not reach statistical significance. On univariate analysis, poor PS was associated with inferior ORR (*p* = 0.03; OR = 0.25, CI = 0.06–0.77) and increased UH (*p* = 0.04; OR = 2.43, CI = 1.05—5.71). There was no significant association between toxicity and any patient characteristic.

**Conclusions:**

We observed inferior OS, ORR, and rates of UH among ICI‐treated RMHNSCC patients with ECOG 2/3. Our findings help frame discussion of therapeutic options in this poor‐risk population.

## Graphical abstract

Relative to those with a performance status of 0‐1, patients with an Eastern Cooperative Oncology Group performance status 2 who received immune checkpoint inhibitors for recurrent/metastatic head and neck cancer have poorer response rates and overall survival as well as increased rates of unplanned hospitalization; no significant difference in toxicity was seen.

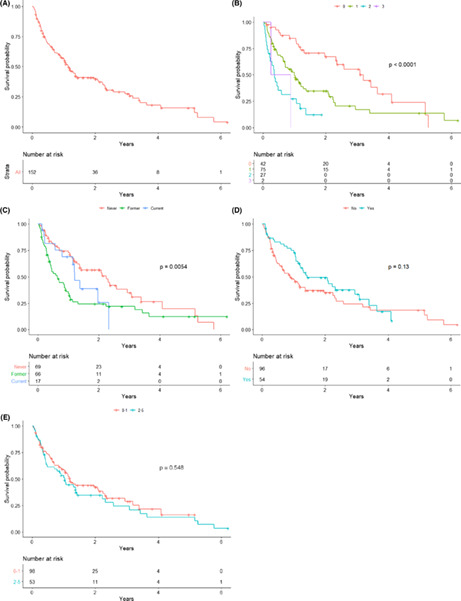

## 1 INTRODUCTION

Head and neck squamous cell carcinomas (HNSCC) generally arise from the mucosa of the oral cavity, oropharynx, larynx, and hypopharynx and account for approximately 4% of new cancer diagnoses in the United States, with more than 66,000 new diagnoses and 14,000 deaths annually.^1^ HNSCC of the oral cavity, larynx and hypopharynx are often related to tobacco/alcohol exposure and are associated with cardiac, pulmonary, and vascular comorbidity, second primary malignancies, socioeconomic, and ethnic disparities. Today, most HNSCCs of oropharynx are related to prior HPV exposure and typically occur in younger, Caucasian males with minimal to no tobacco or alcohol exposure.^2^ Most patients present with locally advanced disease and are treated with curative‐intent, multimodal therapy.^3^ Although prognosis in the HPV+ group is more favorable, outcomes among patients who recur after curative‐intent therapy are suboptimal and survival is measured in months.^4^

Recently, therapeutic options for patients with recurrent and/or metastatic squamous cell carcinomas (RMHNSCC) were transformed by the FDA‐approval of immune checkpoint inhibitors (ICI) targeting programmed cell death protein 1 (PD‐1). These agents confer a survival benefit in the first and second line setting^5^, ^6^ and are generally better tolerated than cytotoxic therapies.^6^, ^7^

Appropriate patient selection when utilizing ICI in RMHNSCC can be a challenging clinical dilemma.^8^ Landmark trials leading to the approval of ICI for RMHNSCC limited enrolment to those with ECOG PS scores of 0–1.^5^, ^6^ However, RMHNSCC is a disease associated with considerable comorbidity, socioeconomic disparity, and heavy symptom burden.^9^ It is not uncommon^10^ for patients with RMHNSCC to have a poor ECOG PS, functional limitations, and disability. Work from our group has demonstrated high rates of hospitalization and end‐of‐life healthcare utilization in this population.^11^ The benefit of ICI in these patients, many of whom are generally poor candidates for trial participation, is not well defined and to date, there have been no studies specifically exploring the relationship between performance status and clinical outcomes in patients with RMHNSCC on ICI.

We examined the pattern of ICI use among RMHNSCC patients at our institution with specific focus on patients with poor PS. We sought to explore the relationship between ECOG PS and oncologic outcomes in addition to obtaining data on the toxicity and unplanned hospitalization (UH) in this group of patients.

## 2 METHODS

We identified a cohort of RMHNSCC patients who were treated with at least one dose of ICI at our institution. Demographic and oncologic data obtained from electronic medical records were recorded in an IRB‐approved database. This project was reviewed and approved by the Fred Hutchinson Cancer Research Center institutional review board.

Performance status was evaluated using the Eastern Cooperative Oncology Group (ECOG) scale and was documented by the patients' oncology team on the day of ICI administration. We defined “poor performance status” as an ECOG PS greater than or equal to 2. Four other patient variables (smoking status, viral association (p16+ or EBV+), prior lines of therapy (which included systemic therapy given in the curative‐intent setting), and patient‐reported race) were also collected for association testing.

Unplanned hospitalization (UH) referred to any unscheduled inpatient admission that occurred between the first dose of ICI and 100 days after the last dose of ICI. Objective response (ORR) was determined both radiographically, as guided by RECIST 1.1 criteria, and clinically, as determined and documented by the patient's primary oncologist; formal RECIST response confirmation was not performed routinely outside of a clinical trial. Treatment‐related toxicity, as graded/reported by the treating physician following initiation of ICI therapy, was also recorded.

### 2.1 Statistical analysis

Overall survival (OS) was defined as time from initiation of ICI to death from any cause.

Hospitalization, toxicity, and objective response were evaluated using logistic regression models**.** OS was estimated using the Kaplan–Meier method; p‐values were from the log‐rank test. A multivariate analysis was performed using Cox regression to explore the relationship between survival, ECOG PS, and other patient factors as noted above. The software “R” was used for all analysis.

## 3 RESULTS

We identified 152 patients treated with ICI at our institution between 2013 and 2019. The average age was 61 (range = 25–90) years. The majority of patients were male (*n* = 124; 81.6%) and White (*n* = 124; 81.6%). The most common primary sites were the oropharynx (*n* = 60; 39.5%) and the oral cavity (*n* = 37; 24.3%). Fifty‐four (35.5%) had p16+ cancers; seven (4.6%) were EBV+. Sixty‐nine (45.4%) were never‐smokers, while 66 (43.4%) were former smokers and the remaining 17 (11.2%) currently smoked. Sixty‐six (43.4%) had a pack‐year history (PYH) >10 years (Table 1).

**TABLE 1:** Patient and tumor characteristics

	*N* = 152 (%)
Age	
<40	6 (3.9%)
40–59	57 (37.5%)
60–79	84 (55.3%)
>80	5 (3.3%)
Self‐identified race	
White	124 (87.3%)
Non‐white	18 (12.7%)
Other or Unknown	10 (6.6%)
Sex	
Male	124 (87.3%)
Female	28 (18.4%)
Smoking status	
Current	17 (11.2%)
Former	66 (43.4%)
Never	69 (45.4%)
Pack year history	
>10 PYH	66 (43.4%)
<10 PYH	85 (56.3%)
Unknown PYH	1 (0.7%)
Site	
Oral cavity	37 (24.3%)
Oropharynx	60 (39.5%)
Nasopharynx	11 (7.2%)
Hypopharynx	7 (4.6%)
Larynx	10 (6.6%)
Salivary gland	1 (0.7%)
Nasal cavity	6 (3.9%)
External auditory canal	3 (2.0%)
Cutaneous SCC	6 (3.9%)
Primary site unknown or not documented	11 (7.2%)
Viral association	
Yes	61 (40.7%)
No	89 (59.3%)
Unknown	2 (1.4%)
Virus type	
HPV+	54 (35.5%)
EBV+	7 (4.6%)
PD‐L1 expression testing	
Yes—>1%	9 (5.9%)
Yes—<1%	1 (0.7%)
No	142 (93.4%)
ECOG	
0	42 (28.8%)
1	75 (51.4%)
2	27 (18.5%)
3	2 (1.4%)
Unknown	6 (3.9%)

At the time of immunotherapy initiation, 42 (28.8%) had an ECOG performance status score of 0, 75 (51.4%) had a PS score of 1, 27 (18.5%) had a PS score of 2, and 2 (1.4%) had a PS score of 3. Six individuals did not have a score recorded and were excluded from our analysis.

Although PD‐L1 expression testing was not routinely performed at the time of study, ten patients (6.6%) did have an available Tumor Proportion Score (TPS) or a Combined Positive Score (CPS); of these, nine (5.9%) had a score ≥1.

The majority of patients (*n* = 144; 94.7%) had received prior therapy; the median number of treatment lines was 1 (range = 0–5). Thirty‐two (21.1%) received XRT within 3 months of ICI. Patients typically received ICI monotherapy (*n* = 118; 77.6%); the remainder received combination therapy, most commonly with ICI + vorinostat while on a clinical trial. Additional treatment characteristics can be seen in Table 2.

**TABLE 2:** Treatment characteristics

	*N* = 152 (%)
Prior curative therapy	
Yes	144 (94.7%)
No	8 (5.3%)
Number of lines of prior TX	
0	25 (16.4%)
1	73 (48.3%)
2	34 (22.5%)
3	13 (8.6%)
4	5 (3.3%)
5	1 (0.7%)
Unknown	1 (0.7%)
XRT within 3 MO of ICI	
Yes	32 (21.1%)
No	120 (78.9%)
ICI monotherapy	
Yes—Single agent	118 (77.6%)
No—Combination therapy	34 (22.4%)
Immunotherapy agent	
Durvalumab	4 (2.6%)
Avelumab	3 (2.0%)
Cemiplimab	6 (3.9%)
Nivolumab	38 (25.0%)
Pembrolizumab	97 (63.8%)
Multiple ICI agents	4 (2.6%)

Unplanned inpatient admission occurred in 57 (38.3%) patients. Patients with poor ECOG PS (2/3 vs. 0/1) experienced higher rates of UH (OR = 2.39, 95% CI = 1.46–4.09; *p* = 0.001). Neither smoking, race, number of prior lines of therapy, nor viral association appeared to affect this variable in our cohort (Table 3). Sixty‐six (44.0%) experienced physician‐documented, CTCAE grade 2 or higher toxicity while on ICI; no patient characteristics appeared to be associated with this outcome.

**TABLE 3:** Univariate models for unplanned hospitalization

	OR	95% confidence interval (low)	95% confidence interval (high)	*p*‐value
ECOG 2–3 vs. 0–1	3.13	1.35	7.53	0.009
Smoking: former vs. never	1.27	0.63	2.58	0.500
Smoking: current vs. never	0.98	0.30	2.91	0.968
Viral association: yes vs. no	0.51	0.24	1.04	0.068
Race: non‐white vs. white	2.27	0.83	6.35	0.109
Number of prior lines of therapy	1.19	0.86	1.66	0.293

**FIGURE 1:**
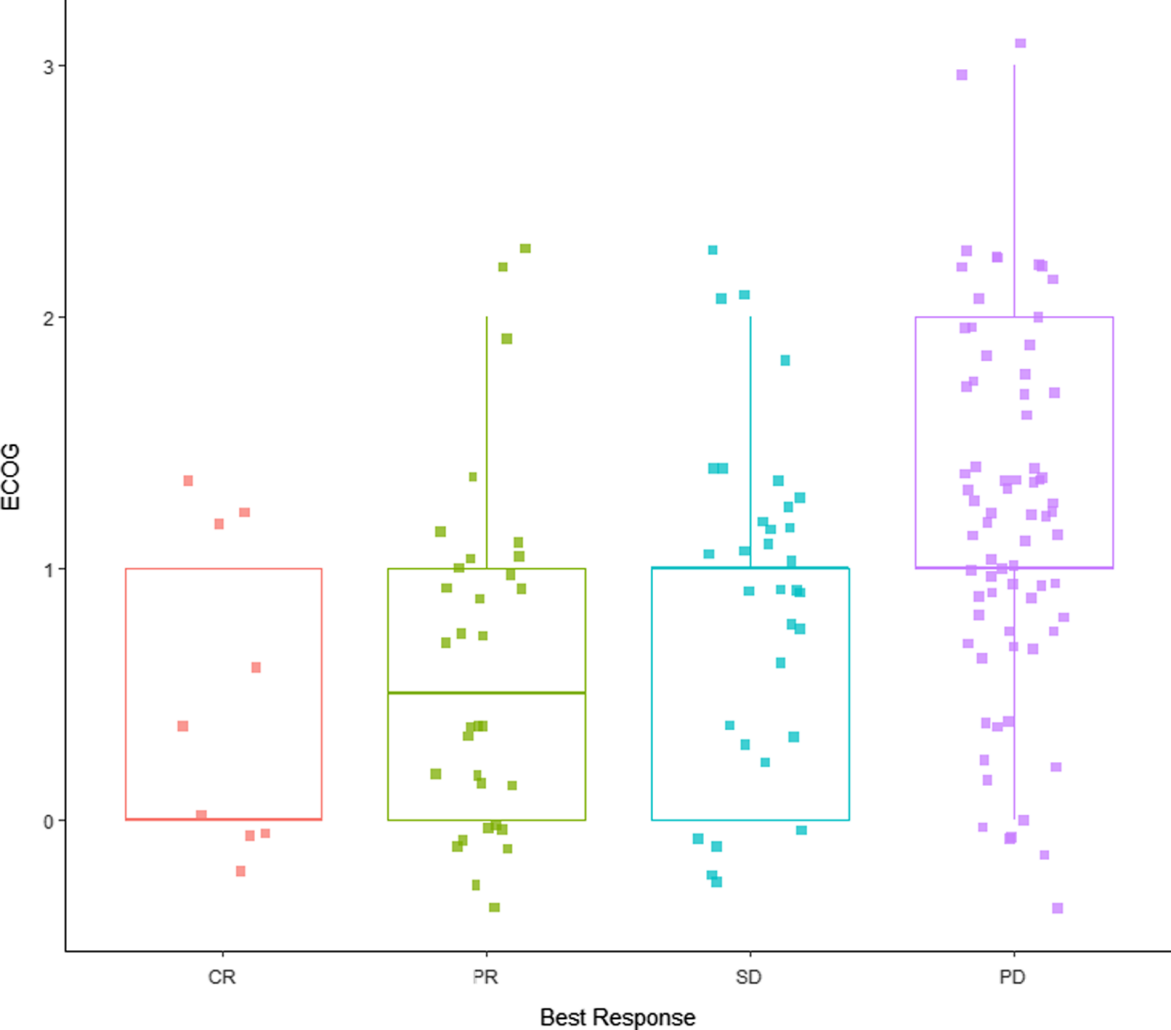
Best response by ECOG PS

Complete response (CR) was achieved in 10 patients (6.7%). Partial response (PR) was seen in 33 (22%) and stable disease (SD) was seen 32 (21.3%). The remaining 75 (50%) experienced progressive disease (PD) as best response; these patients were more likely to have higher ECOG PS at the time of ICI initiation (OR = 0.25, 95% CI = 0.06–0.77, *p* = 0.03; Figure 1, Table 4). Of the patients with ECOG ≥2, 21 had PD, while four had SD, three had PR, and 0 had CR. There was no correlation between number of prior lines of therapy, race or smoking status and treatment response; in contrast patients with virally associated RMHNSCC did appear to experience superior response to therapy (OR = 2.66, 95% CI = 1.28–5.61, *p* = 0.009; Table 5).

**TABLE 4:** Univariate models for response to therapy

	OR	95% confidence interval (low)	95% confidence interval (high)	*p*‐value
ECOG 2–3 vs. 0–1	0.25	0.06	0.77	0.031
Smoking: former vs. never	0.55	0.25	1.19	0.134
Smoking: current vs. never	1.43	0.46	4.25	0.519
Viral association: yes vs. no	2.66	1.28	5.61	0.009
Race: non‐white vs. white	1.04	0.31	2.99	0.948
Number of prior lines of therapy	1.03	0.72	1.45	0.885

**TABLE 5:** Univariate model for overall survival

	HR	95% confidence interval (low)	95% confidence interval (high)	*p*‐value
ECOG 2–3 vs. 0–1	3.07	1.89	4.99	< 0.001
Smoking: former vs. never	1.98	1.30	3.01	0.001
Smoking: current vs. never	1.45	0.74	2.85	0.278
Viral association: yes vs. no	0.73	0.48	1.11	0.136
Race: non‐white vs. white	0.71	0.38	1.36	0.305
Number of prior lines of therapy	1.06	0.88	1.27	0.548

**FIGURE 2:**
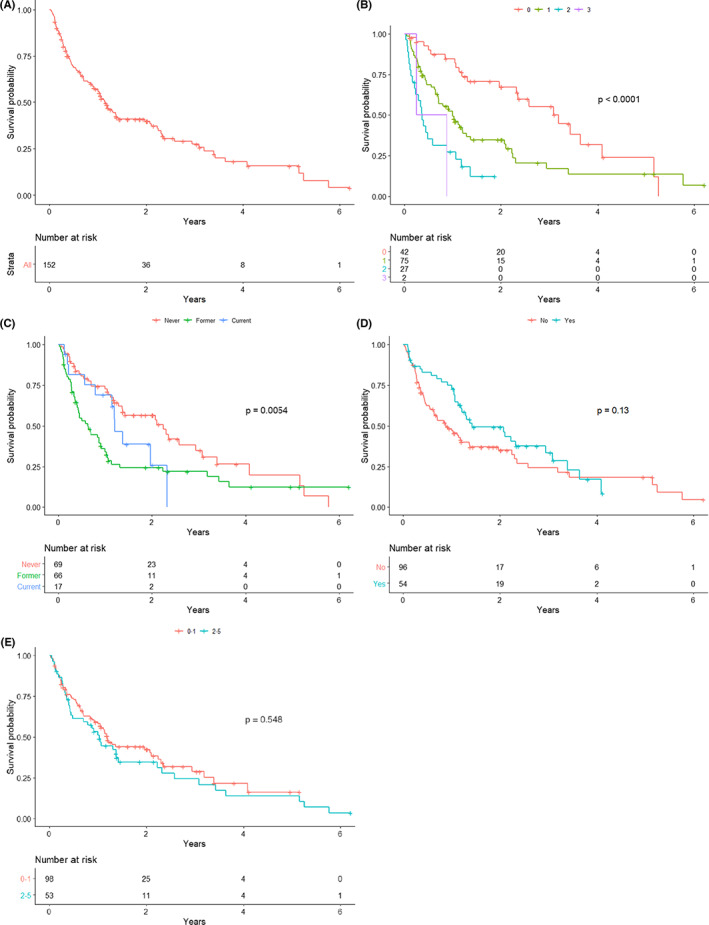
OS in all patients (2A; top left); OS by ECOG PS (2B; top right); OS by smoking status (2C; middle left); OS by viral vs. nonvirally associated disease (2D; middle right); OS by number of prior lines of therapy (2E; bottom)

At the time of our analysis and with a median follow up of 344 days, 137 (90.7%) had documented disease progression and 101 (69.7%) patients had died. ECOG (*p* = <0.0001) and smoking status (*p* = 0.005) were significantly associated with OS. Neither viral association (*p* = 0.136) nor number of prior lines of therapy (*p* = 0.54) appeared to correlate with OS in our cohort of ICI‐treated RMHNSCC (Figure 2).

On multivariable analysis for overall survival, ECOG PS (2/3 vs. 0/1, HR = 3.30, 95% CI 2.01–5.41, *p* < 0.001), as well as smoking status (former vs. never HR = 2.17, 95% CI 1.41–3.35, *p* < 0.001) were independently associated with inferior survival outcomes (Table 6). We additionally performed this multivariable analysis excluding the 17 patients with cutaneous or EBV‐related RMHNCC. In this smaller population, ECOG 2/3 and former smoking continued to be associated with worse OS, (HR = 2.79, 95% CI 1.67–4.64, *p* = 0.001; HR = 2.42, 95% CI 1.53–3.84, *p* = 0.001, respectively), while p16+ (HPV‐related) RMHNSCC was associated with improved OS (HR = 0.61, 95% CI 0.39–0.96, *p* = 0.034) relative to p16‐ malignancy.

**TABLE 6:** Multivariate model for overall survival

	HR	95% confidence interval (low)	95% confidence interval (high)	*p*‐value
ECOG 2–3 vs. 0–1	3.30	2.01	5.41	<0.001
Smoking: former vs. never	2.17	1.41	3.35	<0.001
Smoking: current vs. never	1.54	0.74	3.22	0.248
Viral association: yes vs. no	0.75	0.48	1.16	0.191

## 4 DISCUSSION

Key clinical trials evaluating the efficacy of ICI in RMHNSCC restricted enrolment to patients with an ECOG PS score of 0–1.^5^, ^6^ In clinical practice, however, the favorable toxicity profile of ICI makes these agents attractive for patients with poorer functional status. There is limited literature examining outcomes in the poor PS population, making clinical decision‐making challenging for both patients and providers, who must carefully weigh the risk of adverse effects with any potential survival benefits.

In our cohort of ICI‐treated RMHNSCC, patients had ECOG scores that ranged from 0 to 3 and approximately 20% had a PS of 2 or higher. Not surprisingly,^10^ patients with poor PS experienced significantly worse survival relative to those who initiated ICI with an ECOG PS score of 0–1. These results have been observed in other solid tumors where ICIs represent therapeutic standards and are in‐line with recently published in non‐small cell lung cancer (NSCLC),^12^, ^13^, ^14^, ^15^ as well as bladder cancer.^16^ In the latter study of bladder cancer patients, investigators observed an OS decline among poor PS patients despite an ORR that was comparable to patients with improved performance status. In contrast, our RMHNSCC patients with poor PS experienced an inferior response to therapy; these results are congruent with the observations of Dall'Olio et al., who noted falling ORR in association with PS in patients with NSCLC,^12^ as well as that of Sehgal, who found PS to be an independent risk factor for worse progression‐free survival. Our results are also consistent with previous associations between PS and survival in RMHNSCC patients who receive cytotoxic chemotherapy.^17^

We similarly noted higher rates of unplanned hospitalization (UH) in patients with poor PS. Although the reasons for hospitalization were not captured within our dataset, Correa et al. observed an association between higher PS and worsening dysphagia and large tumor burden in the RMHNSCC population. Admissions may therefore reflect challenges with symptom management (such as bleeding, pain, and issues with swallowing or maintaining a patent airway) that are well documented in advanced disease.^18^ Of note, rates of ICI‐induced toxicity were similar in our cohort irrespective of PS, suggesting differences in rates of UH were unlikely to be attributable to ICI alone. Not unexpectedly, unplanned hospitalizations were more prevalent in the poor PS group. Although difficult to definitively correlate using our dataset, admissions for both disease progression and necessary symptom control likely contributed considerably to this observation and merit consideration when planning therapy for this compromised group.

Although the current body of evidence is small, our results suggest that survival outcomes from landmark trials evaluating ICI efficacy in RMHNSCC may not be generalizable among patients with poor PS. Further study must be done to evaluate this issue as, at present, it is challenging for clinicians to appropriately counsel patients in this population on anticipated outcomes. While largely regarded to be tolerable, ICI is not without risk; 66 (44%) of the patients in our study experienced grade II/III physician‐reported treatment‐related toxicity; in addition, continuing with therapy can be both costly^19^, ^20^ and time consuming. For patients with declining PS, these issues may outweigh the benefits.

Our single‐center, retrospective study had several limitations. The sample size was small and predominantly involved White males; our results may not be generalizable to the broader community of RMHNSCC patients. This was a heterogeneous population reflecting a high‐volume, real‐world clinical practice and included less common and biologically diverse subsets such as EBV‐related nasopharyngeal carcinomas, head and neck cutaneous squamous cell carcinomas, and paranasal sinus squamous cell carcinomas. Oncologic therapy and outcomes that occurred outside our system were not captured in this dataset. In addition, PD‐L1 expression (TPS/CPS score) was not consistently captured and could not be evaluated; as TPS/CPS is known to predict response to therapy/survival,^21^ this represents a possible confounder. Similarly, we did not evaluate the presence of comorbidities that may have contributed to, or existed concomitantly with, a declining PS. Finally, we did not directly compare outcomes in ICI‐RMHNSCC to those who received either cytotoxic chemotherapy or supportive care alone; we therefore cannot comment on whether or not patients who received IC had relative improvement in these outcomes. Future studies would benefit from this analysis.

In conclusion, our results suggest that overall survival, response to therapy, and the incidence of unplanned hospitalizations are inferior among patients with initiating ICI with a poor PS indicating an urgent need to prospectively evaluate interventions in this poor‐risk population.

## CONFLICT OF INTEREST

No conflict of interest or disclosure to report.

## AUTHORS CONTRIBUTION

All authors certify that they have participated in this work. The manuscript was conceptualized and supervised by Rodriguez, with data curation, original draft writing, and review and editing by Chalker. Voutsinas and Wu contributed to methodology and formal analysis. Hwang assisted with data curation. Santana‐Davila, Baik, Lee, Barber, Futran, Houlton, Laramore, Liao, Parvathaneni, Martins, and Eaton all participated in review and editing. This work has been submitted solely to this journal and is not published or under review elsewhere.

## ETHICAL APPROVAL STATEMENT

This project was approved by our institutional review board.

## Data Availability

The data that support the findings of this study are available on request from the corresponding author. The data are not publicly available due to privacy or ethical restrictions.
